# Supplementary material for: Long-term alterations in brain and behavior after postnatal Zika virus infection in infant macaques
Source: Nat Commun. 2020 May 21;11:2534. doi: 10.1038/s41467-020-16320-7 (PMC7242369; doi:10.1038/s41467-020-16320-7)
Supplement: Supplementary file 1 — Supplementary Information [file 41467_2020_16320_MOESM1_ESM.pdf]

Supplementary Information for

Long-term alterations in brain and behavior after postnatal Zika virus infection in infant macaques

Raper et al., Nature Communications, 2020

Supplementary Figure 1. Ventricle enlargement after postnatal ZIKV infection in infant RMs

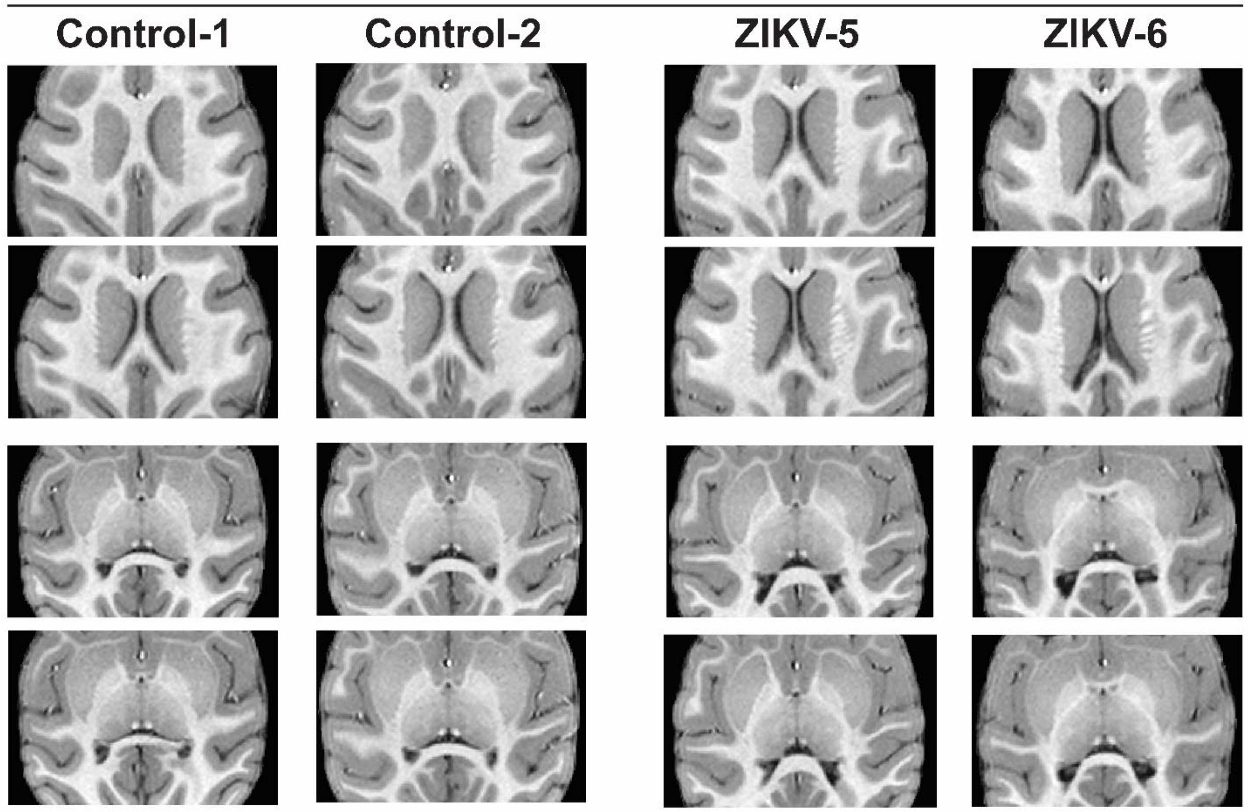

Series of horizontal T1-weighted structural MRI images through the level of the lateral ventricle in control and ZIKV-infected RMs at 12 months of age.

Supplementary Table 1.
Social Behavior with Familiar Partner in Novel Play Cage

Behavior	Subject	Day 1	Day 2	Day 3	Day 4	Day 5	Day 6
Latency to Enter Play Cage (seconds)	Control-1	10.68	1.50	14.45	0.70	1.23	0.87
	Control-2	29.80	18.05	1.50	9.54	0.97	5.07
	ZIKV-5	169.14	143.97	139.40	29.70	2.70	5.51
	ZIKV-6	105.14	208.74	147.91	40.24	10.71	7.54
Percent Time Spent Together with the Familiar Partner	Control-1	93.27	93.24	86.19	86.89	86.87	87.70
	Control-2	93.27	93.24	86.19	86.89	86.87	87.70
	ZIKV-5	90.92	66.37	64.90	61.57	66.49	40.62
	ZIKV-6	90.92	66.37	64.90	61.57	66.49	40.62
Percent Time Spent Away from the Familiar Partner	Control-1	6.73	6.76	13.82	13.11	13.14	12.31
	Control-2	6.73	6.76	13.82	13.11	13.14	12.31
	ZIKV-5	9.08	33.63	34.92	38.43	33.52	59.38
	ZIKV-6	9.08	33.63	34.92	38.43	33.52	59.38
Percent Time Spent Along the Perimeter of the Play Cage	Control-1	18.89	28.25	50.06	65.51	71.64	72.17
	Control-2	4.76	16.99	34.37	46.64	51.27	43.58
	ZIKV-5	97.47	95.65	96.48	94.55	96.95	90.10
	ZIKV-6	96.10	98.52	99.22	93.57	98.08	94.02
Percent Time Spent in Center of the Play Cage	Control-1	80.45	71.12	46.84	34.55	28.41	28.04
	Control-2	95.24	82.96	65.62	53.70	48.77	56.61
	ZIKV-5	2.49	4.28	3.53	5.45	3.15	10.12
	ZIKV-6	1.67	1.48	0.78	6.43	2.30	6.28
Loss of Balance (rate/min)	Control-1	0.085	0.084	0.000	0.043	0.108	0.130
	Control-2	0.021	0.000	0.127	0.043	0.043	0.086
	ZIKV-5	0.063	0.125	0.305	0.318	0.409	0.363
	ZIKV-6	0.124	0.319	0.692	0.404	0.345	0.411
Play Behavior (rate/min)	Control-1	0.076	0.070	0.058	0.07	0.086	0.096
	Control-2	0.019	0.044	0.089	0.04	0.065	0.064
	ZIKV-5	0.027	0.070	0.023	0.02	0.171	0.150
	ZIKV-6	0.033	0.078	0.034	0.01	0.065	0.078
Affiliative Behavior (rate/min)	Control-1	0.296	0.399	0.530	0.494	0.237	0.194
	Control-2	0.806	0.443	0.233	0.215	0.237	0.346
	ZIKV-5	0.169	0.000	0.000	0.000	0.000	0.000
	ZIKV-6	0.435	0.106	0.041	0.085	0.000	0.048
Hostile Behavior (rate/min)	Control-1	0.190	0.168	0.297	0.451	0.538	0.734
	Control-2	0.403	0.274	0.211	0.172	0.409	0.324
	ZIKV-5	0.381	0.852	1.097	2.080	1.785	2.516
	ZIKV-6	0.331	1.084	0.610	1.021	0.711	0.823

Submissive Behavior (rate/min)	Control-1	0.000	0.042	0.000	0.021	0.000	0.022
	Control-2	0.000	0.148	0.021	0.000	0.000	0.022
	ZIKV-5	0.000	0.062	0.081	0.149	0.215	0.194
	ZIKV-6	0.083	0.191	0.102	0.064	0.172	1.016
Anxious Behavior (rate/min)	Control 1	0.296	1.345	0.975	1.117	2.282	1.231
	Control 2	0.021	0.380	0.106	0.172	0.560	0.302
	ZIKV 5	0.042	0.083	0.020	0.085	0.000	0.000
	ZIKV 6	0.041	0.043	0.020	0.043	0.043	0.145
Vocalizations (rate/min)	Control 1	1.648	1.744	1.166	0.988	0.495	1.641
	Control 2	0.085	0.084	0.021	0.129	0.431	0.713
	ZIKV 5	0.909	0.104	0.183	0.021	0.108	0.266
	ZIKV 6	0.455	0.043	0.000	0.043	0.065	0.436
Stereotypies (rate/min)	Control 1	0.000	0.000	0.021	0.000	0.000	0.000
	Control 2	0.713	0.764	0.823	0.151	0.538	0.302
	ZIKV 5	0.266	0.021	0.000	0.000	0.000	0.000
	ZIKV 6	0.103	0.000	0.000	0.000	0.000	0.000

List of behaviors coding during social interaction with a highly familiar partner in a large play cage and the individual values for each subject across the 6 days of testing.

Supplementary Table 2.
Relative Volume of Brain Structures Corrected by Total Brain Volume (TBV)

	Subject			
**Brain Area**	**Control-1**	**Control-2**	**ZIKV-5**	**ZIKV-6**
TBV 12mo (mm^3)	73399.37	84333.91	90258.40	74639.68
Total CSF12mo (mm^3)	11001.10	11709.40	11919.00	10745.40
Total Intracranial Volume (mm^3)	84400.47	96043.31	102177.40	85005.08
Total Lateral Ventricle 12mo (mm^3)	212.53	256.25	340.50	443.24
Total White Matter (WM)/TBV 12mo	29.8880	30.0465	29.6811	28.8612
Total Grey Matter (GM)/TBV 12mo	68.3861	68.2942	68.5905	69.4095
Total corpus callosum/TBV 12mo	1.0800	1.0285	1.1019	0.8875
RT cingulate/TBV 12mo	1.4214	1.4488	1.4252	1.2734
LT cingulate/TBV 12mo	1.4297	1.5371	1.4481	1.3536
Total Amygdala/TBV 12mo	0.7120	0.6441	0.4623	0.5321
Total Hippocampal/TBV 12mo	1.3488	1.2570	1.0513	1.1486
Total Caudate/TBV 12mo	1.6539	1.4213	1.3879	1.6124
Total Putamen/TBV 12mo	2.6849	2.5851	2.2376	2.2612
Total Cerebellum GM/TBV 12mo	5.9876	6.4371	5.3688	5.5499
Total Cerebellum WM/TBV 12mo	3.3330	3.3088	2.8897	3.4664
RT prefrontal GM/TBV 12mo	3.0989	2.7317	3.2436	3.6046
LT prefrontal GM/TBV 12mo	3.3523	3.1865	3.4351	3.6961
RT prefrontal WM/TBV 12mo	0.7397	0.5997	0.7868	0.9645
LT prefrontal WM/TBV 12mo	0.7969	0.8090	0.9260	0.9997
RT frontal GM/TBV 12mo	4.4861	4.6806	4.4204	4.3097
LT frontal GM/TBV 12mo	4.4990	4.4473	4.3250	4.4137
RT frontal WM/TBV 12mo	1.7068	1.8853	1.8290	1.6688
LT frontal WM/TBV 12mo	1.6457	1.7343	1.7029	1.5992
RT parietal GM/TBV 12mo	4.6955	5.2233	5.0329	4.8018
LT parietal GM/TBV 12mo	4.6311	4.8002	4.9160	4.6212

RT parietal WM/TBV 12mo	1.8951	2.0800	2.0286	1.8083
LT parietal WM/TBV 12mo	1.9190	1.9844	1.9667	1.7048
RT occipital GM/TBV 12mo	5.55897	5.1850	6.0033	6.0242
LT occipital GM/TBV 12mo	5.6074	4.7488	5.6875	6.1439
RT occipital WM/TBV 12mo	2.3575	2.5353	2.2570	2.0969
LT occipital WM/TBV 12mo	2.2218	2.2068	2.0854	1.8585
RT temporal auditory GM/TBV 12mo	2.6791	2.6694	2.6412	2.6922
LT temporal auditory GM/TBV 12mo	2.6122	2.8163	2.7998	2.9171
RT temporal auditory WM/TBV 12mo	0.4833	0.5057	0.4976	0.4796
LT temporal auditory WM/TBV 12mo	0.5171	0.5050	0.5606	0.5216
RT temporal visual GM/TBV 12mo	3.6950	3.5612	3.6545	4.1533
LT temporal visual GM/TBV 12mo	4.2582	3.9641	4.1766	4.3187
RT temporal visual WM/TBV 12mo	1.5166	1.4595	1.4967	1.5667
LT temporal visual WM/TBV 12mo	1.5635	1.3299	1.5579	1.5066
RT temporal limbic GM/TBV 12mo	1.7058	1.5913	1.5107	1.3859
LT temporal limbic GM/TBV 12mo	1.4786	1.4933	1.4188	1.2946
RT temporal limbic WM/TBV 12mo	0.2828	0.2940	0.2773	0.2930
LT temporal limbic WM/TBV 12mo	0.2537	0.3032	0.2549	0.2601

TBV = Total Brain Volume; WM = White Matter; GM = Grey Matter; CSF = Cerebrospinal fluid; RT = Right hemisphere; LT = Left hemisphere; 12mo = 12 months of age. The correction used was specific brain region/TBV x 100.

Supplementary Table 3.
Functional Connectivity (r-values) Between Brain Regions of Interest

	Subject			
**Regions of Interest**	**Control-1**	**Control-2**	**ZIKV-5**	**ZIKV-6**
amygdala-amygdala 12mo	0.40446	0.26000	0.20683	0.34853
RT amygdala-hippocampus 12mo	0.37978	-0.05650	-0.06897	0.43040
LT amygdala-hippocampus 12mo	0.42025	0.55852	0.15477	0.28407
RT amygdala-PFC32 12mo	-0.07096	0.12485	-0.11577	0.13632
LT amygdala-PFC32 12mo	0.00266	-0.13709	0.07632	0.03439
RT amygdala-PFC46 12mo	0.00140	0.02658	0.11837	0.11025
LT amygdala-PFC46 12mo	-0.02930	-0.08735	0.22407	0.03536
RT amygdala-OFC11&13 12mo	-0.11172	0.05670	-0.15514	-0.12953
LT amygdala-OFC11&13 12mo	0.11385	-0.04219	0.10252	0.08146
RT amygdala-caudate 12mo	-0.00858	0.00351	0.11016	0.18491
LT amygdala-caudate 12mo	-0.01131	-0.00591	0.19473	0.06599
RT hippocampus-OFC14 12mo	0.10883	0.02029	-0.15121	0.01819
LT hippocampus-OFC14 12mo	0.14742	0.04789	0.00319	0.06395
RT hippocampus-PFC24 12mo	-0.10220	0.09304	0.11650	0.23022
LT hippocampus-PFC24 12mo	0.04504	0.06722	0.13279	0.09732
RT hippocampus-PFC46 12mo	0.19853	0.10712	0.74547	0.07679
LT hippocampus-PFC46 12mo	0.05102	-0.11366	0.150869	0.07209
RT TEO-Putamen 12mo	0.19158	0.10204	-0.02267	0.07764
LT TEO-Putamen 12mo	0.05161	0.10220	0.02511	0.07946
RT TEO-Caudate 12mo	0.05188	-0.03175	0.18854	0.16421
LT TEO-Caudate 12mo	0.00804	0.01805	0.00834	0.12994
RT V1-Putamen 12mo	0.05230	0.07868	0.10253	-0.07566
LT V1-Putamen 12mo	0.11521	0.17489	0.07734	-0.11611

PFC = Prefrontal cortex; OFC = Orbital frontal cortex; V = visual area; RT = Right hemisphere; LT = Left hemisphere; 12mo = 12 months of age

Supplementary Table 4. Behavioral Ethogram

Category and Specific Behaviors	Measurement	Brief Descriptions
Latency to Enter	Duration	Time between the door opening and the animal entering into the social play cage
Location in the Cage		
Perimeter	Duration	Time spent along the walls and outside area of the social play cage
Center	Duration	Time spent on the floor, ceiling, or center pole of the social play cage
Motor Related Behaviors		
Loss of Balance	Frequency	Uncoordinated movement that resulted in a brief loss of balance or footing
Play Behavior	Cumulative Duration	
Exploration	Duration	Use of hands or mouth to nonaggressively grab, hold, or inspect an object, toy, or social play cage
Swing/Jump	Duration	Vigorously jumping or swinging on objects, toys, or part of the social play cage
Distance to Partner		
Together	Duration	Time animals spent in full body contact, proximity (arms length), or within 5 feet of each other
Away	Duration	Time animals spent more than 5 feet apart from each other
Affiliative	Cumulative Frequency	
Touch	Frequency	One animal uses its hand to briefly contact or hold onto the other animal (nonaggressively)
Groom	Frequency	One animal uses its hands or mouth to comb through the fur of the other animal
Follow	Frequency	One animal persistently trails the other, both are actively in motion
Aggression	Cumulative Frequency	
Threat bark	Frequency	Low pitch, high intensity, rasping, guttural
Threat - noncontact	Frequency	Any of the following: open mouth (no teeth exposed), head-bobbing, or ear flapping
Threat - contact	Frequency	Any of the following: bitting, slapping, hitting, or grabbing the other animal
Cage aggression	Frequency	Vigorously slaps, shakes, or slams body against cage
Chase	Frequency	Vigorous and rapid pursuit of the other animal
Submissive	Cumulative Frequency	
Withdrawal	Frequency	Quick, jerky motion away from the stimulus object (jump back)
Present	Frequency	Rigid posture with rump and tail elevated and oriented toward the other animal
Grimace[1]	Frequency	Refracted lips, exposed clenched teeth

Anxiety	Cumulative Frequency	
Scratch	Frequency	Rapid scratch of body with hands or feet
Body shake	Frequency	Shake of the whole body or just head and shoulders region
Tooth grind[1]	Frequency	Repetitive, audible rubbing of upper and lower teeth
Yawn[1]	Frequency	Open mouth widely, exposing teeth
Vocalizations	Cumulative Frequency	
Coo	Frequency	Clear soft pitch and intensity, sounds like “ooooh”
Scream	Frequency	High-pitched vocalization, with extreme high intensity; sounds like “eeeeeee”
Stereotypies	Cumulative Duration	
Pacing	Duration	Repetitive motor pattern around the test cage
Motor stereotypy	Duration	Repetitive, abnormal voluntary or involuntary motor patterns (e.g. swinging, twirling, flipping)

List of all behaviors scored, how they are measured and a brief definition. 1. Behavior that was not exhibited by any animal during observations.
