# Supplementary material for: Long-term alterations in brain and behavior after postnatal Zika virus infection in infant macaques
Source: Nat Commun. 2020 May 21;11:2534. doi: 10.1038/s41467-020-16320-7 (PMC7242369; doi:10.1038/s41467-020-16320-7)
Supplement: Supplementary file 3 — Reporting summary [file 41467_2020_16320_MOESM3_ESM.pdf]

nature research

Corresponding author(s): Ann Chahroudi

Last updated by author(s): Apr 2, 2020

Reporting Summary

Nature Research wishes to improve the reproducibility of the work that we publish. This form provides structure for consistency and transparency in reporting. For further information on Nature Research policies, see Authors & Referees and the Editorial Policy Checklist .

Statistics

For all statistical analyses, confirm that the following items are present in the figure legend, table legend, main text, or Methods section.

n/a	Confirmed	
☐	☒	The exact sample size (*n*) for each experimental group/condition, given as a discrete number and unit of measurement
☐	☒	A statement on whether measurements were taken from distinct samples or whether the same sample was measured repeatedly
☒	☐	The statistical test(s) used AND whether they are one- or two-sided<br>*Only common tests should be described solely by name; describe more complex techniques in the Methods section.*
☒	☐	A description of all covariates tested
☒	☐	A description of any assumptions or corrections, such as tests of normality and adjustment for multiple comparisons
☒	☐	A full description of the statistical parameters including central tendency (e.g. means) or other basic estimates (e.g. regression coefficient) AND variation (e.g. standard deviation) or associated estimates of uncertainty (e.g. confidence intervals)
☒	☐	For null hypothesis testing, the test statistic (e.g. *F*, *t*, *r*) with confidence intervals, effect sizes, degrees of freedom and *P* value noted<br>*Give P values as exact values whenever suitable.*
☒	☐	For Bayesian analysis, information on the choice of priors and Markov chain Monte Carlo settings
☒	☐	For hierarchical and complex designs, identification of the appropriate level for tests and full reporting of outcomes
☒	☐	Estimates of effect sizes (e.g. Cohen's *d*, Pearson's *r*), indicating how they were calculated

Our web collection on statistics for biologists contains articles on many of the points above.

Software and code

Policy information about availability of computer code

Data collection	The Observer XT 14 (Noldus, Inc), Tobii studio software version 3.4.8 (Tobii Technology), Cellsens digital imaging software 1.15 (Olympus)
Data analysis	MATLAB version R2019a (MathWorks), AutoSeg_3.3.2 segmentation package and Nipype for neuroimaging processing

For manuscripts utilizing custom algorithms or software that are central to the research but not yet described in published literature, software must be made available to editors/reviewers. We strongly encourage code deposition in a community repository (e.g. GitHub). See the Nature Research guidelines for submitting code & software for further information.

Data

Policy information about availability of data

All manuscripts must include a data availability statement. This statement should provide the following information, where applicable:

- Accession codes, unique identifiers, or web links for publicly available datasets
- A list of figures that have associated raw data
- A description of any restrictions on data availability

All data used for this paper are available from the authors on request. Supplemental materials contains data tables for social behavior (Supplemental Table 1), structural MRI (Supplemental Table 2), and rs-fMRI (Supplemental Table 3).

Field-specific reporting

Please select the one below that is the best fit for your research. If you are not sure, read the appropriate sections before making your selection.

☒ Life sciences ☐ Behavioural & social sciences ☐ Ecological, evolutionary & environmental sciences

Life sciences study design

All studies must disclose on these points even when the disclosure is negative.

Sample size	Four 12 month old infant macaques (two per group) were used in this study. Nonhuman primate research requires strict adherence to the reduction of animals used in terminal studies, therefore this sample size was determined to provide proof-of-principle to inform future studies on the neurodevelopmental consequences of Zika virus infection after birth.
Data exclusions	No data were excluded from the study. Data tables can be found in supplementary materials.
Replication	To date, no replication study has been performed. This study is an exploratory, proof-of-principle experiment.
Randomization	Infants were pseudo-randomly assigned to either the experimental or control group based on birth date and sex.
Blinding	For social interactions, the experimenter was not blind to the animals group, therefore videos were coded without identifying information about the subject, which was revealed after the coding was complete.

Reporting for specific materials, systems and methods

We require information from authors about some types of materials, experimental systems and methods used in many studies. Here, indicate whether each material, system or method listed is relevant to your study. If you are not sure if a list item applies to your research, read the appropriate section before selecting a response.

Materials & experimental systems

n/a	Involved in the study
☐	☒ Antibodies
☒	☐ Eukaryotic cell lines
☒	☐ Palaeontology
☐	☒ Animals and other organisms
☒	☐ Human research participants
☒	☐ Clinical data

Methods

n/a	Involved in the study
☒	☐ ChIP-seq
☒	☐ Flow cytometry
☐	☒ MRI-based neuroimaging

Antibodies

Antibodies used

Glial fibrillary acidic protein (Agilent Technologies, M0761), NeuN (EMD Millipore, MAB377), caspase 3 (Cell Signaling, 9662), doublecortin (Santa Cruz, SC-8066), Goat anti-doublecortin (1:400 dilution; SantaCruz #sc-8066), and Horse anti-goat secondary antibody (1:200 dilution; Vector labs #BA-9500).

Validation

GFAP (Agilent Technologies, M0761): From the website (https://www.agilent.com/en/product/immunohistochemistry/antibodies-controls/primary-antibodies/glial-fibrillary-acidic-protein-(concentrate)-76524#productdetails Clone 6F2, Labels astrocytes and some CNS ependymal cells. The antibody is a useful aid for classification of tumors of uncertain origin. Immunogen GFAP from human brain, Isotype: IgG1, kappa. Monoclonal mouse antibody provided in liquid form as cell culture supernatant dialysed against 0.05 mol/L Tris-HCl, pH 7.2, and containing 15 mmol/L NaN3. Species : Mouse Anti-Human, Specificity : As demonstrated by immunocytochemistry, the antibody labels GFAP in astrocytes and cells of astrocytic origin.
This antibody in non-human primates: Mavigner et al (2018) Postnatal Zika virus infection causes persistent abnormalities in brain structure, function, and behavior in infant macaques. Science Translational Medicine. 10(435) pii: eaao6975. PMID: 29618564. PMCID: PMC6186170.

NeuN (EMD Millipore, MAB377): From the website (http://www.emdmillipore.com/US/en/product/Anti-NeuN-Antibody-clone-A60,MM_NF-MAB377): MILLIPORE's exclusive monoclonal antibody to vertebrate neuron-specific nuclear protein called NeuN (or Neuronal Nuclei) reacts with most neuronal cell types throughout the nervous system of mice including cerebellum, cerebral cortex, hippocampus, thalamus, spinal cord and neurons in the peripheral nervous system including dorsal root ganglia, sympathetic chain ganglia and enteric ganglia. Developmentally, immunoreactivity is first observed shortly after neurons have become postmitotic, no staining has been observed in proliferative zones. The immunohistochemical staining is primarily localized in the nucleus of the neurons with lighter staining in the cytoplasm. Anti-NeuN Antibody, clone A60 detects level of NeuN and has been published and validated for use in FC, IC, IF, IH, IH(P), IP and WB. This antibody in non-human primates Burke et al., 2009. Neuronal reduction in frontal cortex of primates after prenatal alcohol exposure. Neuroreport 20(1). 13-7; Mortazavi et al., 2017. A survey of white matter neurons at the gyral crowns and sulcal depths in the rhesus monkey. Front Nauroanat 11:69.

Caspase 3 (Cell Signaling, 9662) From the website (https://www.cellsignal.com/products/primary-antibodies/caspase-3-antibody/9662) Caspase-3 (CPP-32, Apoptain, Yama, SCA-1) is a critical executioner of apoptosis, as it is either partially or totally responsible for the proteolytic cleavage of many key proteins, such as the nuclear enzyme poly (ADP-ribose) polymerase (PARP) (1). Activation of caspase-3 requires proteolytic processing of its inactive zymogen into activated p17 and p12 fragments.

Cleavage of caspase-3 requires the aspartic acid residue at the P1 position (2). Fernandes-Alnemri, T. et al. (1994) J Biol Chem 269, 30761-4. Nicholson, D. W. et al. (1995) Nature 376, 37-43. Polyclonal antibodies are produced by immunizing animals with a synthetic peptide corresponding to residues surrounding the cleavage site of human caspase-3. Antibodies are purified by protein A and peptide affinity chromatography. Species Reactivity: Human, Mouse, Rat, Monkey.

This antibody in non-human primates: Mavigner et al (2018) Postnatal Zika virus infection causes persistent abnormalities in brain structure, function, and behavior in infant macaques. Science Translational Medicine. 10(435) pii: eaao6975. PMID: 29618564. PMCID: PMC6186170.

Doublecortin (Santa Cruz, SC-8066; http://datasheets.scbt.com/sc-8066.pdf): This particular antibody has over 270 pubmed-listed references and we have successfully used this in non-human primates (see Curtis et al., 2014). From Santa Cruz website: Doublecortin (C-18) is an affinity purified goat polyclonal antibody raised against a peptide mapping at the C-terminus of Doublecortin of human origin. Doublecortin (C-18) is recommended for detection of Doublecortin of mouse, rat and human origin by Western Blotting (starting dilution 1:200, dilution range 1:100-1:1000), immunoprecipitation [1-2 µg per 100-500 µg of total protein (1 ml of cell lysate)], immunofluorescence (starting dilution 1:50, dilu- tion range 1:50-1:500), immunohistochemistry. References: De La Rosa-Prieto, C., et al. 2015. Olfactory and cortical projections to bulbar and hippocampal adult-born neurons. Front. Neuroanat. 9: 4; Rao and Shetty, 2004. Efficacy of doublecortin as a marker to analyse the absolute number and dendritic growth of newly generated neurons in the adult dentate gyrus. Eur J Neurosci 19(2): 234-46.; Curtis et al., 2014. Reduction of pyramidal and immature hippocampal neurons in pediatric simian immunodeficiency virus infection. Neuroreport 25(13): 973-8.

Animals and other organisms

Policy information about studies involving animals; ARRIVE guidelines recommended for reporting animal research

Laboratory animals	Four 12 month old female infant Indian rhesus macaques (Macaca mulatta) from the Yerkes National Primate Research Center colony.
Wild animals	No wild animals were used in this study.
Field-collected samples	No field-collected samples were used in this study. Macaques born at the Yerkes National Primate Research Center and were housed indoors on a 12hour light-dark cycle (7am-7pm). Macaques were socially housed in pairs with visual and auditory contact with another pair of the same age. all were fed a diet of Purina Primate Chow, Old World Monkey formulation, supplemented with daily fruits and vegetables. Water was provided ad libitum.
Ethics oversight	This study was conducted in strict accordance with U.S. Department of Agriculture regulations and the recommendations in the Guide for the Care and Use of Laboratory Animals of the National Institutes of Health, approved by the Emory University Institutional Animal Care and Use Committee, and conducted in an AAALAC accredited facility.

Note that full information on the approval of the study protocol must also be provided in the manuscript.

Magnetic resonance imaging

Experimental design

Design type	Structural and resting-state functional (rs-fMRI) MRI scans
Design specifications	8 T1 images, 3 T2 images, 2x15 min rs-fMRI EPI sequences with an additional short reverse-phase encoding scan were collected
Behavioral performance measures	No behavioral task was conducted in the MRI. MRI scans were performed under anesthesia and only resting-state fMRI was analyzed.

Acquisition

Imaging type(s)	Structural and Functional
Field strength	3T
Sequence & imaging parameters	Structural: High-resolution T1-weighted MRI scans were acquired for volumetrics and registration of the functional scans using a three-dimensional magnetization-prepared rapid gradient echo (3D-MPRAGE) parallel image sequence [repetition time/echo time (TR/TE) = 2600/3.46 ms; field of view (FOV), 116 mm × 116 mm; voxel size, 0.5 mm3 isotropic; eight averages] with GeneRalized Autocalibrating Partially Parallel Acquisitions (GRAPPA) acceleration factor of R = 2. T2-weighted MRI scans were collected using a 3D fast spin-echo sequence (TR/TE = 3200/373 ms; FOV, 128 mm × 128 mm; voxel size, 0.5 mm3 isotropic; three averages) with GRAPPA (R = 2) to aid with registration and delineation of anatomical borders.<br>Functional: BOLD-weighted functional images were collected using a single-shot echo-planar imaging (EPI) sequence (2X400 volumes; TR/TE = 2060/25 ms, 2 × 15 min; voxel size, 1.5 mm3 isotropic) after the T1-MRI scan. An additional short, reverse-phase encoding scan was also acquired for unwarping susceptibility-induced distortions in the EPI images using previously validated methods.
Area of acquisition	Whole brain

Diffusion MRI ☐ Used ☒ Not used

Preprocessing

Preprocessing software	sMRI: Scans were processed using AutoSeg_3.0.2 segmentation package.<br>rs-fMRI: All imaging data was pre-processed similarly for normalization and comparison purposes using the FMRIB Software Library (FSL, Oxford, UK) (Smith et al. 2004; Woolrich et al. 2009)), 4dfp tools, and an in-house pipeline built using Nipype (Gorgolewski et al. 2011) with modifications of published methods (Fair et al. 2007; Fair et al. 2009; Fair et al. 2012; Iyer et al. 2013; Miranda-Dominguez et al. 2014a), including some adapted for the rhesus monkey brain (Miranda-Dominguez et al. 2014b; Godfrey et al. 2018).
Normalization	sMRI: AutoSeg uses the ANTS registration tool (Grossman et al. 2008; Wang et al. 2014) to register each atlas/template image to the subjects' brain image. In order to do this, ANTS uses a cross-correlation similarity metric and a symmetric diffeomorphic deformation model to preserve the geometric properties of the subject image even if large distortions are needed for the registration.<br>rs-fMRI: After the EPI functional time series were concatenated and rigid-body co-registered to the subject's averaged T1-weighted structural image, the T1-weighted structural images were transformed to conform to the age-specific (12months) T1-weighted rhesus infant brain structural MRI atlas/template developed by our group (Shi et al. 2017), using non-linear registration methods in FSL (FNIRT).
Normalization template	sMRI: The 12months UNC-Emory infant rhesus atlas (publicly available at: https://www.nitrc.org/projects/macaque_atlas/, Shi et al. 2017) was used, which is a template of scans acquired at 12 months of age on 40 infant rhesus monkeys from the YNPRC social colony, balanced by sex and social rank.<br>rs-fMRI: The 12months UNC-Emory infant rhesus atlas was previously registered to the 112RM-SL atlas (publicly available at: http://brainmap.wisc.edu/monkey.html) in F99 space (McLaren et al. 2009; McLaren et al. 2010).
Noise and artifact removal	sMRI: Image processing steps included averaging the T1 and the T2 images to improve signal-to-noise ratio, and intensity inhomogeneity correction, using N4-ITK bias field correction.<br>rs-fMRI: As part of the noise and artifact reducing procedures, after file conversion, functional imaging series were: 1) unwarped using a reverse phase-encoding distortion correction method (TOPUP correction, Anderson et al. 2003), 2) slice-time corrected (for the even vs. odd slice intensity differences due to interleaved acquisition), 3) motion-corrected (rigid body motion correction within-run, linear registration from EPI to T1, and nonlinear registration from T1 to template applied all in one resampling step), and 4) normalized (signal normalization to a whole brain mode value gradient of 1000 was done to scale the BOLD values across subjects at an acceptable range to perform the rest of preprocessing steps). Additional pre-processing steps included: 1) functional signal detrending; 2) nuisance regressor removing (i.e. regression of rigid body head motion parameters in 6 directions, regression of the global whole-brain signal, regression of the ventricular and white matter functional signal (averaged from a ventricle- and a white matter mask, respectively), and regression of the first-order derivatives for the whole brain, ventricular and white matter signals); and 3) temporal low-pass filtering ($f < 0.1Hz$) via second order Butterworth filter (Fair et al. 2007; Fair et al. 2009; Fair et al. 2012; Miranda-Dominguez et al. 2014b). Global signal regression (GSR) was applied based on current literature that highlights the importance of removing systematic artifacts in the data, such as global artifacts originated not just from movement, but from respiratory and other physiological noises (Yan et al. 2013; Burgess et al. 2016; Ciric et al. 2017; Nalci et al. 2017; Power et al. 2017)
Volume censoring	Analyses were conducted with the removal of frames with displacement (FD) value greater than 0.2 mm (Power et al. 2012; Power et al. 2014), using Matlab. This censoring caused no volume removal in either one of the subjects.

Statistical modeling & inference

Model type and settings	Proof-of-Principle study with small sample size, thus direct statistical analyses were not appropriate.
Effect(s) tested	Proof-of-Principle study with small sample size, thus direct statistical analyses were not appropriate.

Specify type of analysis: ☐ Whole brain ☒ ROI-based ☐ Both

Anatomical location(s)	ROIs were defined based on the combined Lewis and Van Essen (2000) and Markov et al. (2014) published anatomical parcellations mapped onto the cortical surface of the UNC-Emory rhesus infant atlases (Shi et al. 2017), registered to the F99 space. The left and right amygdala label maps were manually drawn by experts using cytoarchitectonic maps in the existing UNC-Wisconsin adolescent atlas (Styner et al. 2007), and then propagated to the UNC-Emory rhesus infant atlases in F99 space, using deformable registration via ANTS (Shi et al. 2017). Each ROI was then manually edited in the infant macaque F99 atlas following established anatomical landmarks (Paxinos et al. 1999; Saleem and Logothetis 2012) for neuroanatomical accuracy and to avoid 1) ROI overlap and 2) voxels with signal dropout (non-brain tissue signal).
Statistic type for inference (See Eklund et al. 2016)	Eklund et al, 2016 does not apply because this study was did not conduct voxelwise analyses
Correction	MRI analyses were primarily descriptive due to the small sample size, thus did not require multiple comparisons correction

Models & analysis

n/a	Involved in the study
☐	☒ Functional and/or effective connectivity
☒	☐ Graph analysis
☒	☐ Multivariate modeling or predictive analysis

Functional and/or effective connectivity

The rs-fMRI BOLD time series were correlated ROI by ROI for each subject. For this, the time course of the BOLD signal was averaged across the voxels within each ROI, and then correlated with the time course of the other ROIs.
